# Anti-Staphylococcal Biofilm Effects of a Liposome-Based Formulation Containing Citrus Polyphenols

**DOI:** 10.3390/antibiotics13040318

**Published:** 2024-03-30

**Authors:** Diletta Mazzantini, Mariacristina Massimino, Marco Calvigioni, Virginia Rossi, Francesco Celandroni, Antonella Lupetti, Giovanna Batoni, Emilia Ghelardi

**Affiliations:** Department of Translational Research and New Technologies in Medicine and Surgery, University of Pisa, Via San Zeno 37, 56123 Pisa, Italy; diletta.mazzantini@unipi.it (D.M.); mariacristina.massimino@gmail.com (M.M.); marco.calvigioni@med.unipi.it (M.C.); v.rossi33@studenti.unipi.it (V.R.); francesco.celandroni@unipi.it (F.C.); antonella.lupetti@unipi.it (A.L.); giovanna.batoni@unipi.it (G.B.)

**Keywords:** polyphenols, liposomes, staphylococci, biofilm

## Abstract

Biofilms are surface-associated microbial communities embedded in a matrix that is almost impenetrable to antibiotics, thus constituting a critical health threat. Biofilm formation on the cornea or ocular devices can lead to serious and difficult-to-treat infections. Nowadays, natural molecules with antimicrobial activity and liposome-based delivery systems are proposed as anti-biofilm candidates. In this study, the anti-biofilm activity of a formulation containing citrus polyphenols encapsulated in liposomes was evaluated against *Staphylococcus aureus* and *Staphylococcus epidermidis*, the most common agents in ocular infections. The formulation activity against planktonic staphylococci was tested by broth microdilution and sub-inhibitory concentrations were used to evaluate the effect on biofilm formation using the crystal violet (CV) assay. The eradicating effect of the preparation on mature biofilms was investigated by the CV assay, plate count, and confocal laser scanning microscopy. The product was bactericidal against staphylococci at a dilution of 1:2 or 1:4 and able to reduce biofilm formation even if diluted at 1:64. The formulation also had the ability to reduce the biomass of mature biofilms without affecting the number of cells, suggesting activity on the extracellular matrix. Overall, our results support the application of the used liposome-encapsulated polyphenols as an anti-biofilm strategy to counter biofilm-associated ocular infections.

## 1. Introduction

Eyes are anatomical parts responsible for vision and characterized by self-mechanisms of defense against external microbes, including the presence of an ocular microbiota and the production of antimicrobial compounds diffused by continuous tear flow [1]. However, microbial colonization of ocular devices (e.g., contact lenses), exogenous microbial invasion through ocular trauma or surgery, dry eye conditions, lacrimal duct obstruction, and intraocular incursion from other anatomical sites can lead to ocular infections [2].

Keratitis, blepharitis, uveitis, and endophthalmitis are among the infectious diseases that can affect eyes, potentially reducing/impairing vision and compromising quality of life [3]. These diseases are mainly caused by bacteria, with the Gram-positive *Staphylococcus epidermidis* and* Staphylococcus aureus* as the predominantly involved species [4,5,6]. Although at a lower rate than bacteria, fungi, viruses, and parasites can also be responsible for ocular infections [7,8]. 

Some staphylococcal ocular infections (e.g., keratitis, post-cataract endophthalmitis) have been shown to be associated with the presence of biofilms, particularly in people wearing contact lenses or undergoing surgery with the placement of ocular devices (e.g., intraocular lenses, orbital implants, scleral buckles, conjunctival plugs) [9,10]. In addition, biofilms can be directly formed on the cornea, leading to keratitis [11]. Biofilms are microbial communities embedded in an extracellular polymeric matrix constituted by exopolysaccharides, surfactants, lipids, proteins, and DNA, commonly known as extracellular polymeric substances (EPSs) [12,13]. These structures are formed by a multi-step process that requires bacterial adhesion to surfaces, synthesis and secretion of EPS, cell multiplication with biofilm maturation, and dispersal of planktonic microbes to colonize new environments [13]. Since the biofilm matrix is almost impenetrable to antibiotics, antiseptics, and immune cells and houses persister cells, biofilms are often responsible for persistent and recalcitrant infections that are very difficult to treat [11,14]. In addition, biofilms can contribute to the spreading of drug resistance, thus constituting a serious threat for human health [12]. Therefore, many efforts have been made in developing novel anti-biofilm strategies, and some options, including quorum-sensing (QS) inhibitors, bacteriophages, and natural molecules with antimicrobial and anti-biofilm activities, have been proposed [13,15,16,17]. In addition, particular emphasis has been placed on the development of innovative delivery systems for antimicrobial encapsulation, which can cross the biofilm matrix, releasing their cargo directly on biofilm-embedded cells [18]. In this view, liposomes were shown to be promising nano-vehicles for drugs in biofilm-associated infections, and several antimicrobials have been liposome-encapsulated to increase their stability, delivery, and antimicrobial activity [18,19,20,21].

Recently, a formulation containing a mixture of citrus polyphenols encapsulated in liposomes was introduced on the Italian market as an antiseptic for the ocular surface [22,23]. Polyphenols are natural molecules produced as secondary metabolites by plants and constituted by one or more phenolic rings with hydroxyl groups. Besides their known antioxidant, anti-inflammatory, and beneficial properties, these molecules exert broad-spectrum antimicrobial activity, mainly attributable to their ability to destabilize membrane potential and affect pH gradient, resulting in the disruption of the plasma membrane [24,25]. Polyphenols were also shown to affect DNA synthesis, QS expression, energy metabolism, and the activity of bacterial enzymes [15,24,25,26,27]. Some polyphenols, particularly flavonoids, phenolic acids, and tannins, were additionally shown to exert anti-biofilm activity in vitro. Nevertheless, their activities are different depending on the tested molecule [15]. 

Since both polyphenols and liposomes are now believed to be potential innovative anti-biofilm options [13,15,16,17,18,21], in this study we spotlight the anti-biofilm activity of the liposomal polyphenol formulation, focusing on its ability to affect biofilm formation and eradicate mature biofilms. The anti-biofilm activity of the liposome-based product was tested against *S. aureus* and *S. epidermidis*, which are the most common agents of ocular infections and known to be biofilm-proficient [4,5,6]. 

## 2. Results

### 2.1. Activity against Planktonic Staphylococci 

The analyzed formulation (Oftasecur Ocular Spray, hereafter referred to as OS) showed antibacterial activity against both susceptible and antibiotic-resistant staphylococci, with Minimal Inhibitory Concentration (MIC) values ranging from a product dilution of 1:2 (for *S. aureus* ATCC 6538, *S. epidermidis* CI-1, and *S. epidermidis* ATCC 35984) to 1:4 (for *S. aureus* ATCC 43300 and *S. epidermidis* CI-2). Then, the Minimal Bactericidal Concentration (MBC) of the formulation was evaluated. No colonies were obtained by seeding aliquots from wells containing OS at the MIC values, thus indicating that the formulation is bactericidal against all the tested microbes. When planktonic staphylococci were assayed for levofloxacin susceptibility, *S. aureus* ATCC 6538, *S. aureus* ATCC 43300, *S. epidermidis* ATCC 35984, and *S. epidermidis* CI-1 were found to be susceptible to increased exposure to this drug (MIC: 0.25 mg/L) on the basis of the EUCAST Breakpoint Tables version 14.0 for *Staphylococcus* spp. [28]. In contrast, *S. epidermidis* CI-2 were found to be levofloxacin resistant (MIC: 8 mg/L).

### 2.2. Inhibitory Effect on Biofilm Formation

With the aim to select sub-inhibitory OS concentrations to be used in biofilm formation assays, growth curves in the presence of OS dilutions from 1:8 to 1:64 were established. The product dilutions 1:8 and 1:16 were found to impact the growth of each strain (Figure S1). In contrast, the OS dilutions 1:32 and 1:64 did not affect the growth kinetics of the tested strains, resulting in growth curves completely overlapping those obtained for the growth control (Figure S1). Based on these results, the OS dilutions 1:32 and 1:64 were selected for the biofilm inhibition assays.

**Figure 1:**
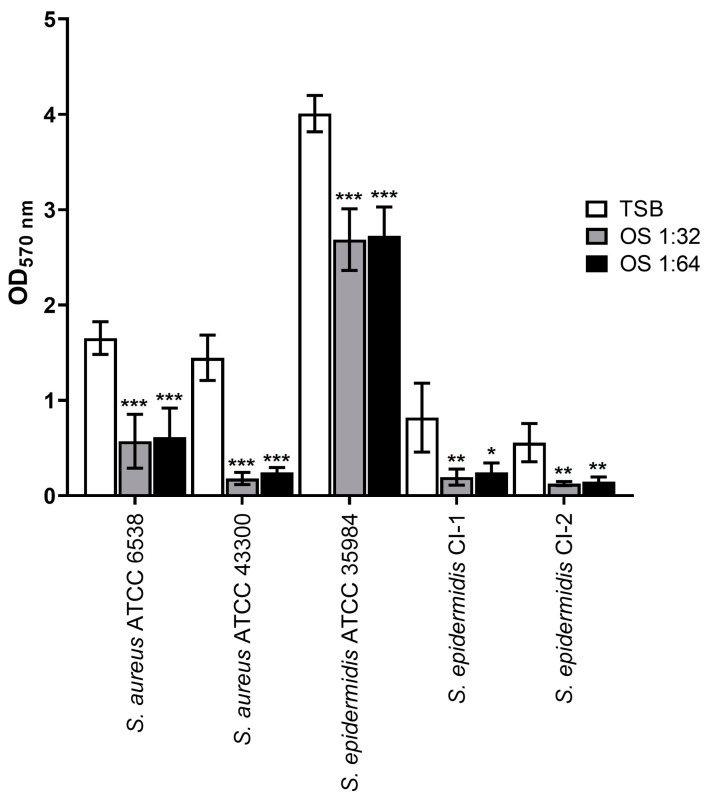
Effect of sub-inhibitory concentrations of OS on biofilm formation. White bars: TSB (control); grey bars: OS dilution 1:32; black bars: OS dilution 1:64. OD_570nm_: optical density measured at 570 nm. * *p* < 0.05; ** *p* < 0.01; *** *p* < 0.001.

*Staphylococcus* strains were inoculated in the presence of product dilutions 1:32 and 1:64 and tested for their ability to form biofilms in comparison to the untreated controls. As shown in Figure 1, the presence of OS significantly reduced the biofilm biomasses compared to the respective controls in TSB (*p* < 0.05, *p* < 0.01, and *p* < 0.001 depending on strain and OS dilution). Overall, these findings indicate that the liposomal polyphenol mixture reduces biofilm formation by the tested *Staphylococcus* strains.

### 2.3. Eradicating Effect on Mature Biofilms

To test the ability of the formulation to eradicate mature staphylococcal biofilms, 24-hour-old biofilms were treated with undiluted OS. Biofilms treated with levofloxacin (concentrations ranging from 0.5 to 512 µg/mL) were also analyzed for comparison (Table S1). *S. epidermidis* CI-2 was excluded from the treatment with levofloxacin, since it is resistant to this antibiotic. 

**Figure 2:**
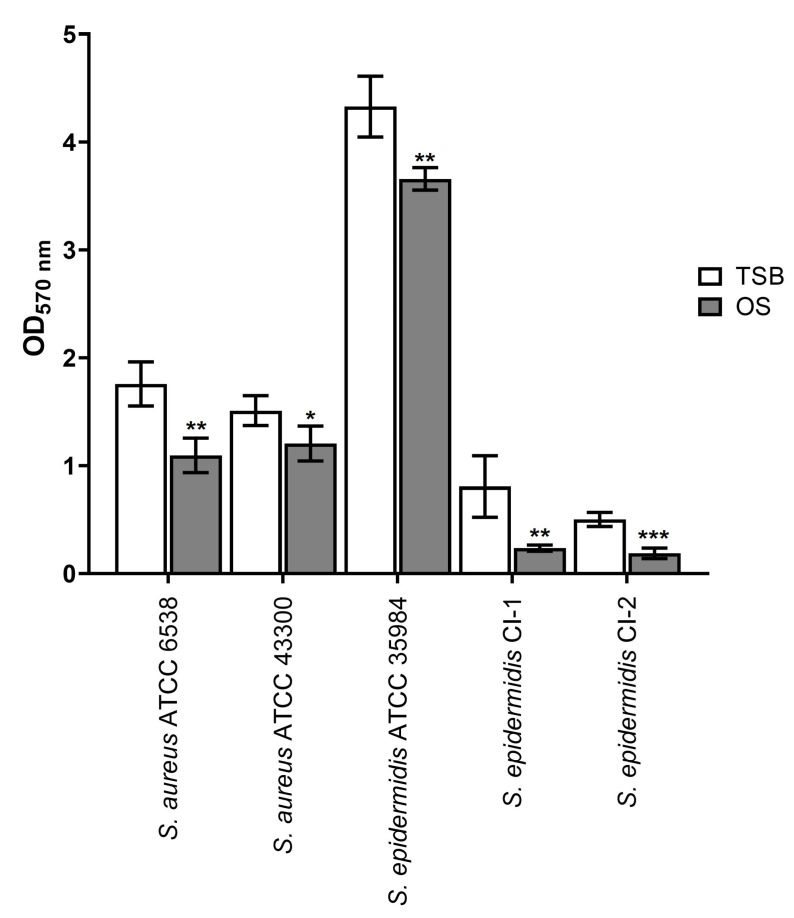
Effect of OS on mature staphylococcal biofilms. White bars: biofilm formed in TSB (control); grey bars: biofilm treated with OS. OD_570nm_: optical density measured at 570 nm. * *p* < 0.05; ** *p* < 0.01: *** *p* < 0.001.

As shown in Figure 2, the treatment with OS significantly reduced the biomass of biofilms formed by all strains compared to controls in TSB (*p* < 0.05 for *S. aureus* ATCC 43300, *p* < 0.01 for *S. aureus* ATCC 6538, *S. epidermidis* ATCC 35984, *S. epidermidis* CI-I, and *p* < 0.001 for *S. epidermidis* CI-2). 

The most prominent eradicating effect was obtained against the biofilms formed by *S. epidermidis* CI-1 and CI-2, with calculated percentages of eradication of 67.83 ± 11.45% and 52.00 ± 11.38%, respectively. As regards *S. epidermidis* CI-1, the anti-biofilm activity of OS was significantly greater than that of levofloxacin used at the highest concentration of 512 µg/mL (*p* < 0.05; Table 1). 

The lowest biofilm-eradicating activity of OS was obtained against *S. epidermidis* ATCC 35984 (percentage of eradication of 15.19 ± 6.31%), but this effect was comparable to that of levofloxacin at concentrations ranging from 0.5 to 128 µg/mL (Table 1). For *S. aureus* ATCC 6538 and ATCC 43300, the calculated percentages of eradication due to OS were 37.87 ± 1.91% and 20.28 ± 6.60%, respectively. These activities were comparable to that of levofloxacin at concentrations ranging from 1 to 128 µg/mL and from 0.5 to 32 µg/mL, respectively (Table 1). Taken together, these findings indicate that OS can reduce the biomass of preformed staphylococcal biofilms, thus exerting biofilm-eradicating activity. 

**Figure 3:**
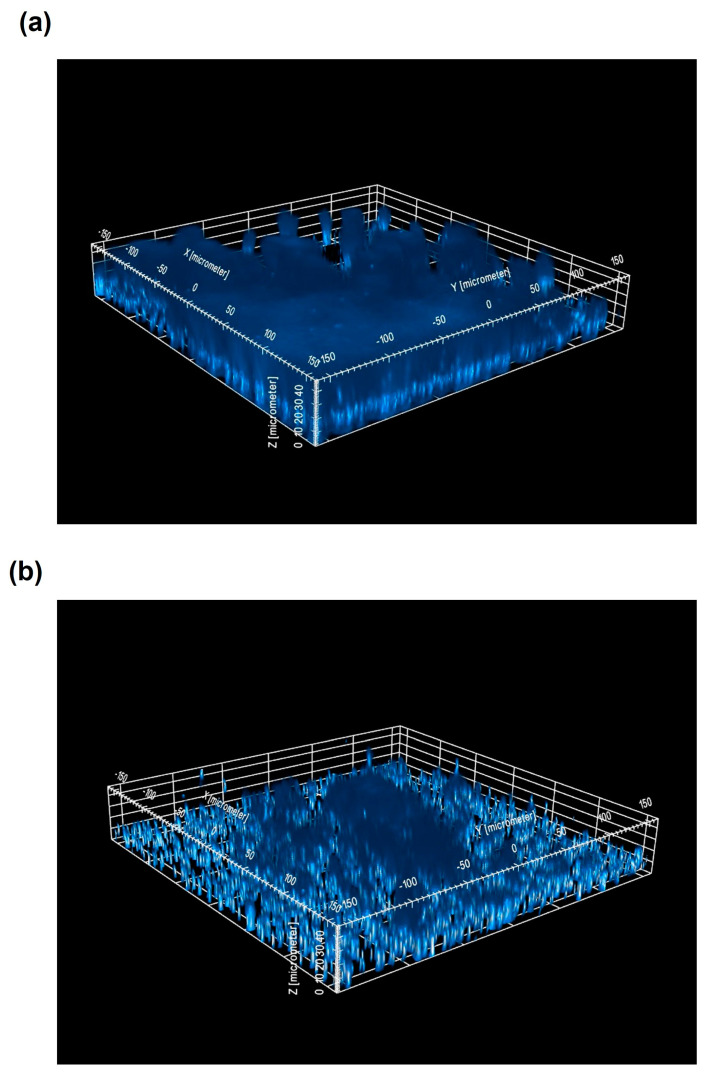
Activity of undiluted OS on pre-formed biofilms of *S. epidermidis* CI-2, which was selected as the representative strain. Three-dimensional reconstruction of the DAPI-stained biofilm of *S. epidermidis* CI-2 in TSB (control) (**a**) and treated with OS (**b**). Data from a representative experiment are shown. For each dimension, the scale bar is indicated in brackets.

The reduction in the OD_570nm_ values observed after treatment with the liposomal mixture could be due to an effect of the product on cell viability or on the extracellular biofilm matrix. With the aim to test whether OS had killing activity on biofilm cells, the number of viable biofilm-associated bacteria was determined by plate count. No differences in the number of CFU/mL were observed when biofilms were treated with OS compared to the TSB control for all the tested strains (*p* > 0.05, Table S2). To confirm this result, biofilm cells were labeled with DAPI, a cell-permeant DNA binding fluorescent stain, and observed by Confocal Laser Scanning Microscopy (CLSM). No differences in the number of cells and in the mean intensity of DAPI emitted by cells was evidenced for the tested strains in OS-treated biofilms compared to the untreated controls (*p* > 0.05; Table S2). Nevertheless, when observed by CSLM, the treated biofilms appeared to be always thinner than the untreated counterparts (Figure 3). 

Overall, these results suggest that the anti-biofilm activity of OS is attributable to a decrease in the biofilm extracellular matrix.

### 2.4. Activity of OS in Combination with Levofloxacin

To test whether synergic activity of the product could be evidenced when associated with levofloxacin, pre-formed staphylococcal biofilms were simultaneously treated with OS and levofloxacin, and the biofilm biomass was quantified by the crystal violet (CV) assay. These assays were conducted on *S. aureus* ATCC 6538 and *S. epidermidis* CI-1, since biofilms formed by these strains were the most susceptible to OS or levofloxacin treatment. For each strain, three levofloxacin concentrations with at least 35% biofilm-eradicating activity were selected (8, 16, and 32 µg/mL for *S. aureus* ATCC 6538 and 128, 256, and 512 µg/mL for *S. epidermidis* CI-1). Although a significant reduction in the OD_570 nm_ values of biofilms treated with the antimicrobials alone or in combination compared to positive control wells was observed (Table 2, *p* < 0.001), the formulation did not show synergic anti-biofilm effects when combined with levofloxacin at different concentrations. In fact, no significant reduction in the OD_570nm_ values was found when *S. aureus* ATCC 6538 biofilms were simultaneously treated with OS and levofloxacin, regardless of drug concentration (*p* > 0.05 compared to values obtained with OS and levofloxacin alone). Although the OD_570nm_ values obtained with drug combinations against *S. epidermidis* CI-1 biofilms were significantly lower than those of levofloxacin alone (*p* < 0.01 or *p* < 0.001), no differences were observed when compared to those of OS. Overall, these findings indicate that the product does not increase levofloxacin activity.

## 3. Discussion

Staphylococcal biofilms represent a critical issue in device-related eye infections and in ocular diseases like keratitis [9,11]. In fact, most *S. aureus* and *S. epidermidis* ocular isolates have been shown to be biofilm-proficient on abiotic surfaces, including ocular prostheses and contact lenses, and on ex vivo human corneas [29,30,31,32,33,34,35,36].

Considering the resistance of microbial communities organized in biofilms to conventional antibiotic treatment, which makes biofilm-related infections challenging to treat, new anti-biofilm options are urgently needed [12,13,14,15,16,17,18]. The main aim of this in vitro study was to evaluate the anti-staphylococcal biofilm potential of a commercial formulation constituted by a mixture of citrus polyphenols encapsulated in liposomes. The product displays antimicrobial activity and negligible cytotoxicity on corneal and conjunctival epithelial cells when used undiluted [37,38,39], but no information on its anti-biofilm effect is available. 

Before evaluating the anti-biofilm potential of the formulation, we tested its antimicrobial activity against planktonic staphylococci, including antibiotic-susceptible and -resistant strains. We show that the product is active and bactericidal against all the tested microbes in vitro. The bactericidal activity of OS is mainly attributable to the ability of the mixture of polyphenols contained in liposomes to disrupt the bacterial plasma membrane, thus leading to cell death [24,25]. 

When the effect of the product on biofilm formation was evaluated, we found that sub-inhibitory concentrations of OS significantly reduced the formation of staphylococcal biofilms. This finding could have an important implication in vivo, with OS potentially preventing biofilm formation, thus reducing the development of biofilm-associated infections. As regards the anti-biofilm action of the product, we can only speculate that the contained polyphenols affect the regulatory network involved in the first stage of biofilm formation (e.g., the attachment), thus reducing biofilm establishment. In fact, the anti-biofilm activity of polyphenols was mainly attributed to their influence on QS or other global regulatory systems, without a direct effect on the viability of biofilm cells [15,21,40].

Microbial biofilms are more resistant to antimicrobial treatments than planktonic bacteria, and massive dosages of antibiotics are required to eradicate biofilm-related infections [14,27,37]. For this reason, we decided to use the undiluted formulation (i.e., two-fold the MIC of planktonic *S. aureus* ATCC 6538, *S. epidermidis* ATCC 35984, and *S. epidermidis* CI-1, and four-fold the MIC of planktonic *S. aureus* ATCC 43300 and *S. epidermidis* CI-2) for testing its eradicating activity on pre-formed biofilms. In addition, different concentrations of levofloxacin were tested in parallel to compare the anti-biofilm activity of OS with that of an antibiotic widely used in ophthalmology [41,42]. Similar to other fluoroquinolones, levofloxacin displays better anti-biofilm effects than other antibiotics and represents the recommended therapy for keratitis [41,42,43].

Herein, we show that the liposome-based formulation is active in reducing the biomass of mature staphylococcal biofilms. The eradicating effect of the product was found to be comparable to that of high levofloxacin concentrations. This finding is appealing, since administration of the product to patients with ocular biofilms could promote the reduction of the biofilm biomass, increasing the possibility of its eradication when combined with an antibiotic therapy.

Interestingly, the finding that biofilms treated with OS show lower absorbances and thickness compared to the untreated ones, but not a reduced number of biofilm-associated bacteria, lead us to hypothesize an activity of OS on the biofilm extracellular matrix. In this view, since liposomes were shown to infiltrate the biofilm matrix [44,45,46], we speculate that some liposomes are able to cross the matrix, releasing polyphenols in proximity of the embedded cells and thus affecting biofilm regulatory mechanisms (e.g., QS system) influencing EPS synthesis/degradation. A similar effect on the biofilm matrix was previously observed for other polyphenols [47,48]. Other studies will be required for clarifying the effect of OS on biofilm matrices.

Combinations of different antimicrobials can lead to synergic anti-biofilm effects, which can increase the rate of biofilm eradication compared to single drugs [49]. For this reason, we evaluated the potential eradicating effect of the formulation in combination with levofloxacin. Unfortunately, no increase in biofilm eradication was obtained, thus indicating that OS does not exert a synergic anti-biofilm effect when combined with this antibiotic. Other studies are needed to screen the anti-biofilm effect of the product in combination with other antimicrobials.

In conclusion, considering the relevance of staphylococci in ocular and biofilm-related infections, this study supports the efficacy of the tested formulation in preventing biofilm formation and reducing the biomass of pre-formed biofilms. These properties highlight the potential use of liposome-encapsulated citrus polyphenols as promising prophylactic agents to prevent the emergence of ocular biofilms and as valid antibiotic adjuvants for treating biofilm-associated infections of the eye.

## 4. Materials and Methods

### 4.1. Bacterial Strains, Culture Conditions, and Chemicals

In this study, *Staphylococcus aureus* ATCC 6538, *S. aureus* ATCC 43300, *Staphylococcus epidermidis *ATCC 35984, and two clinical isolates of *S. epidermidis* (named CI-1 and CI-2) were used. *S. epidermidis* CI-2 was used in a previous study [31], while *S. epidermidis* CI-1 was obtained from a specimen collected at the Clinical Microbiology Laboratory of the Pisa University Hospital and identified by Matrix-Assisted Laser Desorption/Ionization-Time of Flight Mass Spectrometry [50].

Strains were previously characterized for antibiotic susceptibility, and *S. aureus* ATCC 43300,* S. epidermidis* ATCC 35984, and *S. epidermidis* CI-2 were defined as multi-drug resistant, since they showed resistance to at least one drug belonging to at least three different antibiotic classes (i.e., oxacillin, gentamycin, and erythromycin) [51]. All the strains were propagated on Mueller–Hinton Agar (MHA; Thermo Fisher Scientific, Waltham, MA, USA) at 37 °C for 24 hours (h). For susceptibility assays by broth microdilution, cation-adjusted Mueller–Hinton Broth (CAMHB; Thermo Fisher Scientific, Waltham, MA, USA) was used [52]. Growth curves and biofilm assays were performed using Tryptone Soy Broth (TSB, Thermo Fisher Scientific, Waltham, MA, USA), since this medium is commonly used for biofilm assays using *Staphylococcus* strains [53,54]. OS (lot number: 071221; expiry: December 2024) was kindly provided by OFFHEALTH S.p.A. (Florence, Italy). The spray contained 0.2% Biosecur, 0.15%, hypromellose, 1%, phospholipids S80, boric acid, sodium tetraborate decahydrate, sodium chloride, and distilled water. Levofloxacin powder was purchased from Thermo Fisher Scientific (Waltham, MA, USA) and dissolved in a suitable solvent as indicated by the manufacturer to a concentration of 10 mg/mL.

### 4.2. MIC and MBC

OS and levofloxacin were two-fold serially diluted in CAMHB in 96-well microplates (Carlo Erba, Milan, Italy) to obtain a final volume of 100 µL per well. The dilutions of the product used in the assays ranged from 1:2 to 1:4096, while levofloxacin concentrations ranged from 0.031 to 64 mg/L. Bacterial inocula were prepared by suspending a colony freshly grown on MHA in sterile saline solution (0.85% NaCl) to a density of 0.5 McFarland (corresponding to ~1–2 × 10^8^ CFU/mL). Bacteria were diluted in CAMHB to a final concentration of ~5 × 10^5^ CFU/mL [28], and 100 µL was inoculated in wells of flat 96-well microplates (Carlo Erba, Milan, Italy). In parallel, wells containing bacteria in CAMHB and sterile CAMHB were used as positive and negative controls, respectively. Microplates were incubated at 35 ± 1 °C for 18 ± 2 h, and the MICs of OS and levofloxacin were determined [52]. The MIC was defined as the lowest concentration of antimicrobials that completely inhibited visible growth and was determined following the EUCAST reading guide for broth microdilution version 5.0 [55]. For levofloxacin, susceptibility categories (S, susceptible, standard dosing regimen; I, susceptible, increased exposure; R, resistant) were defined based on EUCAST Breakpoint Tables version 14.0 for *Staphylococcus* spp. [28]. Determination of OS MBC was performed by plating 100 µL of the suspensions taken from wells at the MIC and at concentrations of OS higher than the MIC. Plates were incubated at 37 °C for 24 h, and the MBC was defined as the lowest concentration of the formulation killing at least 99.9% of viable microbes.

### 4.3. Growth Curves

Bacteria were grown overnight in 5 mL of TSB broth at 37 °C, and 200 µL was inoculated in 20 mL of fresh TSB (as control) and in 20 mL of TSB containing OS at different sub-inhibitory concentrations (product dilution 1:8, 1:16, 1:32, and 1:64). Cultures were incubated for up to 24 h at 37 °C in an orbital shaker (SYC-2102A; Crystal Technology & Industries, Addison, TX, USA). Growth curves were determined by measuring the OD_600 nm_ using a BioSpectrophotometer^®^ (Eppendorf, Milan, Italy) at 2 h time intervals up to 8 h and at 24 h.

### 4.4. Biofilm Inhibition Assay

*Staphylococcus* strains were grown overnight in 5 mL of TSB broth at 37 °C and diluted 1:100 in fresh TSB (control) and in TSB containing OS at sub-inhibitory concentrations (product dilution 1:32 and 1:64). Bacterial suspensions (200 µL) were dispensed into flat-bottom polystyrene 96-well microplates (Carlo Erba, Milan, Italy). Wells containing TSB alone and TSB supplemented with OS at dilutions 1:32 and 1:64 were also included as negative controls. Microplates were incubated statically at 37 °C for 24 h. At the end of incubation, the medium containing non-adherent bacteria was removed, and wells were washed three times with phosphate buffered saline (PBS; 1 M KH_2_PO_4_, 1 M K_2_HPO_4_, 5 M NaCl, pH 7.2). Biofilms were stained by adding 0.3% of CV and incubating for 30 minutes (min) at room temperature. Then, wells were washed three times with distilled water. and bound CV was solubilized with 96% ethanol for 15 min. Biofilm biomass was estimated by measuring the OD_570nm_. For each strain, the OD_570nm_ values were adjusted by subtracting the OD_570nm_ obtained for negative controls.

### 4.5. Eradication of Mature Biofilms

Bacterial strains were grown overnight in 5 mL of TSB broth at 37 °C and diluted 1:100 in fresh TSB. Suspensions (200 µL/well) were dispensed into flat-bottom polystyrene 96-well microplates (Carlo Erba, Milan, Italy), and plates were incubated statically at 37 °C for 24 h to allow biofilm formation. Wells containing TSB without bacteria were used as negative controls. After incubation, supernatants were removed and wells were inoculated with 200 µL of undiluted OS or with 200 µL of fresh TSB as a control. In parallel, the biofilm-eradicating effect of levofloxacin was tested using antibiotic concentrations ranging from 0.5 to 512 µg/mL. Microplates were incubated statically for an additional 24 h. Biofilm staining and biomass determination were performed as described in Section 4.4. For each strain, the OD_570nm_ values were adjusted by subtracting the OD_570nm_ obtained for negative controls. The rate of biofilm eradication was calculated as follows: biofilm eradication (%) = [(control OD_570nm_ − treated sample OD_570nm_)/control OD_570nm_] × 100 [56].

In parallel, a count of biofilm-associated bacteria was performed. Briefly, biofilms were detached from wells by scraping with a sterile tip and transferred to 1 mL of PBS. Suspensions were vigorously vortexed for 30 s (s), sonicated for 30 s using a water bath sonicator (Ultrasonic cleaner, VWR), and vortexed for an additional 30 s. After being diluted in PBS, aliquots were seeded on agar plates for CFU counts [57].

### 4.6. CLSM Analysis of Staphylococcal Biofilms

Pre-formed staphylococcal biofilms (24 h old) were treated with OS for 24 h (as described in Section 4.5). Supernatants were removed, and biofilms were fixed by adding 1 mL of 2% (*w*/*v*) paraformaldehyde (PFA, Sigma-Aldrich, St. Louis, MI, USA) and incubating at 4 °C for 16 h in a dark room. After PFA removal, wells were washed three times with 200 µL of PBS. Then, cellular DNA was marked by adding DAPI (1 μg/mL) in a dark room and incubating for 4 h at room temperature. DAPI was removed, and wells were covered with 200 µL of PBS. DAPI-stained biofilms were observed using the Operetta CLS High-Content Analysis System (PerkinElmer Inc., Boston, MA, USA), analyzing 25 fields per well and acquiring 25 plane confocal images (from −1 to 35 µm) per field at 40× magnification. Images were then analyzed by Harmony software (Version 5.2, Perkin Elmer Inc., Boston, MA, USA), and the number of cells and the DAPI intensity of each plane were quantified.

### 4.7. Analysis of Synergism

To assess the possible synergy between OS and levofloxacin, 24 h old *S. aureus* ATCC 6538 and *S. epidermidis* CI-1 biofilms were washed three times with PBS and treated with 200 µL of OS containing different levofloxacin concentrations (ranging from 8 to 32 µg/mL for *S. aureus* ATCC 6538 and from 128 to 512 µg/mL for *S. epidermidis* CI-1) for an additional 24 h [58]. Wells inoculated with TSB, OS, and levofloxacin alone were included as controls. After incubation, supernatants were discarded, and biofilms were washed three times with PBS. Biofilm staining with CV, biomass quantification, and calculation of biofilm eradication (%) were performed as described above.

### 4.8. Statistical Analysis

At least three independent biological replicates with two technical replicates each were performed. Quantitative data were expressed as the mean ± standard deviation (SD). All statistical analyses and graphs were performed using GraphPad Prism (version 8.0.2, Dotmatics, Boston, MA, USA). Depending on the experiment, the Student’s *t*-test for unpaired data or the One-Way ANOVA for independent data followed by a Tukey post hoc test for multiple comparisons was used. A two-tailed *p*-value (*p*) < 0.05 was considered significant.

## Figures and Tables

**Table 1:** Percentages of eradication obtained with different levofloxacin concentrations. Values (%) are expressed as the mean ± standard deviation.

Levofloxacin Concentration (µg/mL)	*S. aureus* ATCC 6538	*S. aureus* ATCC 43300	*S. epidermidis* ATCC 35984	*S. epidermidis *CI-1
0.5	18.81 ± 6.80	11.13 ± 3.83	11.26 ± 5.80	7.07 ± 3.04
1	27.91 ± 8.78	11.42 ± 5.29	12.26 ± 5.13	14.50 ± 3.77
2	31.80 ± 10.32	12.36 ± 4.19	12.46 ± 4.04	18.00 ± 10.50
4	36.36 ± 4.67	27.33 ± 7.57	13.10 ± 4.19	21.35 ± 4.34
8	41.48 ± 6.02	28.93 ± 6.09	13.49 ± 2.74	23.44 ± 5.16
16	43.77 ± 7.11	33.26 ± 6.29	17.70 ± 5.93	33.67 ± 11.23
32	46.79 ± 9.00	34.29 ± 4.01	17.83 ± 1.43	34.85 ± 4.82
64	52.97 ± 4.51	35.41 ± 8.16	18.68 ± 3.42	35.38 ± 6.09
128	54.27 ± 10.62	35.49 ± 4.07	24.85 ± 12.65	35.43 ± 6.35
256	96.43 ± 0.985	36.98 ± 5.83	45.94 ± 3.15	37.75 ± 12.99
512	98.52 ± 0.468	37.22 ± 6.68	65.91 ± 4.92	46.30 ± 6.29

**Table 2:** Biomass of biofilms (OD_570nm_) treated with OS and/or levofloxacin at different concentrations. The % of eradication is indicated in brackets. Values are expressed as the mean ± standard deviation.

	*S. aureus* ATCC 6538	*S. epidermidis *CI-1
TSB ^a^	1.99 ± 0.14	1.10 ± 0.09
OS	1.25 ± 0.14 (37.48 ± 5.58)	0.374 ± 0.03 (65.83 ± 2.00)
Levofloxacin 8 ^b^ or 128 ^c^ µg/mL	1.161 ± 0.14 (41.36 ± 10.82)	0.719 ± 0.04 (33.93 ± 8.35)
Levofloxacin 16 ^b^ or 256 ^c^ µg/mL	1.086 ± 0.04 (45.37 ± 5.08)	0.694 ± 0.02 (36.36 ± 5.60)
Levofloxacin 32 ^b^ or 512 ^c^ µg/mL	1.046 ± 0.19 (47.52 ± 9.52)	0.594 ± 0.02 (45.58 ± 4.45)
OS + levofloxacin 8 ^b^ or 128 ^c^ µg/mL	1.16 ± 0.06 (41.46 ± 6.46)	0.39 ± 0.05 (64.42 ± 2.09)
OS + levofloxacin 16 ^b^ or 256 ^c^ µg/mL	1.02 ± 0.02 (48.62 ± 4.77)	0.380 ± 0.06 (64.92 ± 7.94)
OS + levofloxacin 32 ^b^ or 512 ^c^ µg/mL	1.019 ± 0.01 (48.75 ± 3.99)	0.372 ± 0.04 (65.84 ± 4.74)

^a^ Control. ^b^ Levofloxacin concentration used for *S. aureus* ATCC 6538. ^c^ Levofloxacin concentration used for *S. epidermidis *CI-1.

## Data Availability

Datasets generated during the current study will be made available by the corresponding author on reasonable request.

## Supplementary Materials

The following supporting information can be downloaded at: https://www.mdpi.com/article/10.3390/antibiotics13040318/s1, Figure S1: Staphylococcal growth in the presence of sub-inhibitory Oftasecur concentrations.; Table S1: Effect of levofloxacin on mature biofilm biomass; Table S2: Quantification of biofilm-embedded cells (plate count and CLSM analysis) and of DAPI intensity emitted by cells (CLSM analysis).
