# Supplementary material for: Anti-Staphylococcal Biofilm Effects of a Liposome-Based Formulation Containing Citrus Polyphenols
Source: Antibiotics (Basel). 2024 Mar 30;13(4):318. doi: 10.3390/antibiotics13040318 (PMC11047357; doi:10.3390/antibiotics13040318)
Supplement: Supplementary file 1 [file antibiotics-13-00318-s001.zip › antibiotics-2892984-supplementary.pdf]

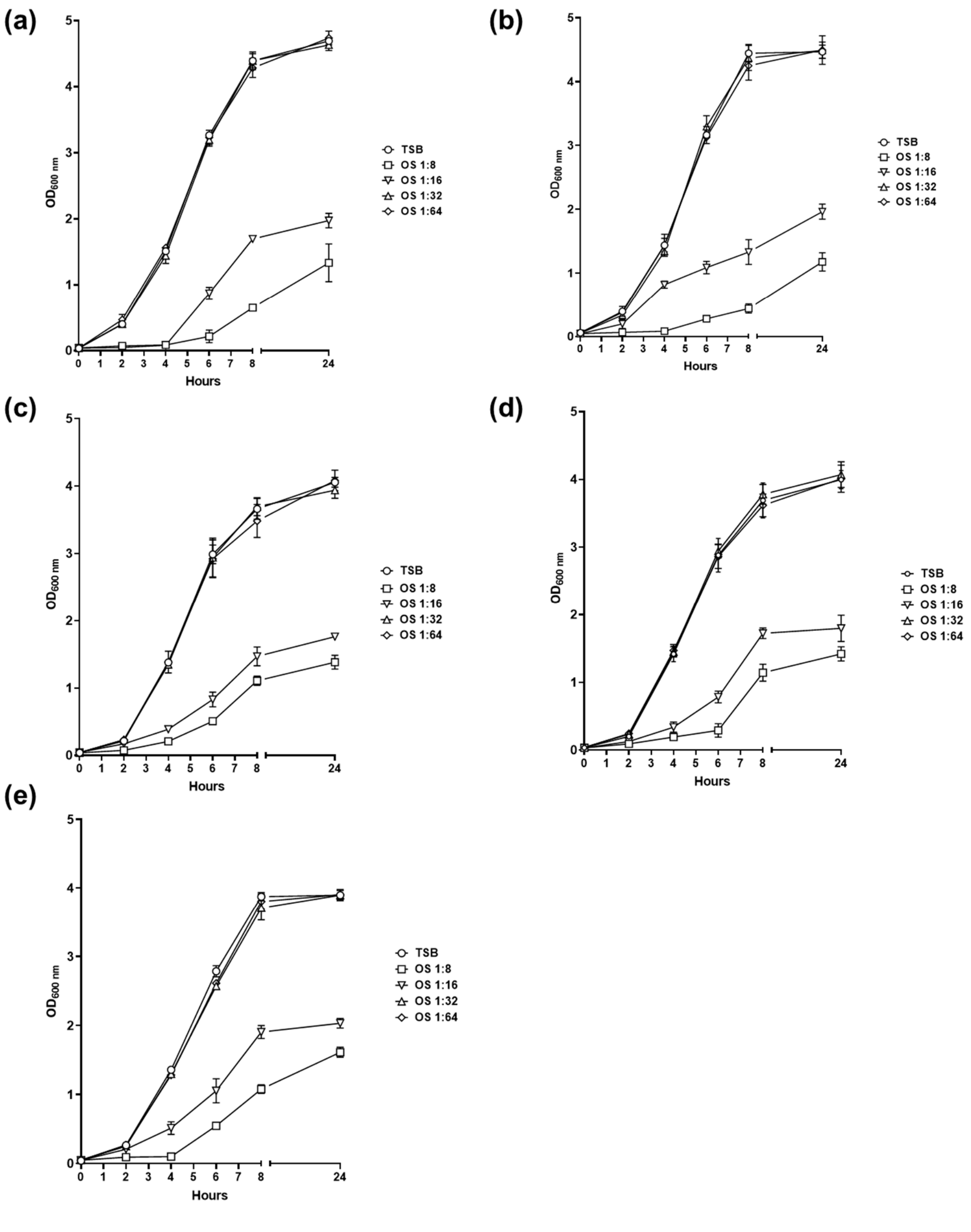

Figure S1. Staphylococcal growth in the presence of sub-inhibitory OS concentrations. Effect of sub-inhibitory OS concentrations (product dilution 1:8, 1:16, 1:32, and 1:64) on the growth of *S. aureus* ATCC 6538 (a), *S. aureus* ATCC 43300 (b), *S. epidermidis* ATCC 35984 (c), *S. epidermidis* CI-1 (d), and *S. epidermidis* CI-2 (e). For each strain, growth in TSB without OS was monitored as control.

Table S1. Effect of levofloxacin on mature biofilm biomass. Values ($OD_{570\ nm}$) are expressed as the mean ± standard deviation.

**Levofloxacin concentration (µg/mL)**	***S. aureus* ATCC 6538**	***S. aureus* ATCC 43300**	***S. epidermidis* ATCC 35984**	***S. epidermidis* CI-1**
0[a]	1.763 ± 0.220	1.474 ± 0.133	4.850 ± 0.125	0.815 ± 0.078
0.5	1.424 ± 0.149	1.312 ± 0.150	4.303 ± 0.272	0.755 ± 0.051
1	1.260 ± 0.111	1.310 ± 0.188	4.259 ± 0.341	0.694 ± 0.038
2	1.193 ± 0.164	1.294 ± 0.155	4.242 ± 0.115	0.664 ± 0.063
4	1.129 ± 0.218	1.072 ± 0.155	4.217 ± 0.291	0.639 ± 0.045
8	1.029 ± 0.155	1.044 ± 0.070	4.200 ± 0.226	0.622 ± 0.056
16	0.982 ± 0.079	0.978 ± 0.039	3.987 ± 0.206	0.609 ± 0.019
32	0.925 ± 0.089	0.967 ± 0.085	3.984 ± 0.051	0.533 ± 0.084
64	0.822 ± 0.030	0.944 ± 0.041	3.941 ± 0.089	0.529 ± 0.093
128	0.792 ± 0.122	0.949 ± 0.078	3.648 ± 0.649	0.524 ± 0.043
256	0.062 ± 0.020	0.924 ± 0.036	2.621 ± 0.136	0.500 ± 0.056
512	0.026 ± 0.008	0.920 ± 0.057	1.655 ± 0.223	0.435 ± 0.032

[a]TSB control.

Table S2. Quantification of biofilm-embedded cells (plate count and CLSM analysis) and of DAPI intensity emitted by cells (CLSM analysis). Data are expressed as the mean ± standard deviation.

	**Plate count (CFU/mL)**		**Cells count by CLSM (cell/well)**		**Intensity of DAPI emitted by cells (CLSM)**	
**Bacterial strain**	**Control TSB**	**OS**	**Control TSB**	**OS**	**Control TSB**	**OS**
S. aureus ATCC 6538	$4.13 \pm 0.072 \times 10^7$	$4.26 \pm 0.40 \times 10^7$	$2.78 \pm 0.86 \times 10^7$	$3.48 \pm 0.31 \times 10^7$	$8.67 \pm 2.64 \times 10^4$	$4.32 \pm 3.03 \times 10^4$
S. aureus ATCC 43300	$4.80 \pm 0.91 \times 10^7$	$3.98 \pm 0.66 \times 10^7$	$3.14 \pm 0.15 \times 10^7$	$3.50 \pm 0.18 \times 10^7$	$3.66 \pm 1.64 \times 10^4$	$2.55 \pm 0.34 \times 10^4$
S. epidermidis ATCC 35984	$5.53 \pm 0.68 \times 10^7$	$5.24 \pm 0.34 \times 10^7$	$3.62 \pm 0.41 \times 10^7$	$2.28 \pm 0.76 \times 10^7$	$7.15 \pm 1.06 \times 10^5$	$4.82 \pm 1.22 \times 10^5$
S. epidermidis CI-1	$5.53 \pm 0.65 \times 10^7$	$4.35 \pm 0.91 \times 107$	$3.32 \pm 0.41 \times 10^7$	$3.36 \pm 0.23 \times 10^7$	$2.91 \pm 1.18 \times 10^4$	$3.69 \pm 0.88 \times 10^3$
S. epidermidis CI-2	$1.85 \pm 0.42 \times 10^7$	$1.59 \pm 0.25 \times 10^7$	$3.30 \pm 0.077 \times 10^7$	$3.57 \pm 0.79 \times 10^7$	$2.32 \pm 0.49 \times 10^4$	$3.03 \pm 0.61 \times 10^4$
